# Comparative proteomic analysis of transition of *saccharomyces cerevisiae* from glucose-deficient medium to glucose-rich medium

**DOI:** 10.1186/1477-5956-10-40

**Published:** 2012-06-12

**Authors:** Bennett J Giardina, Bruce A Stanley, Hui-Ling Chiang

**Affiliations:** 1 Department of Cellular and Molecular Physiology, Penn State University College of Medicine, 500 University Drive, Hershey, PA, 17033, USA; 2 Section of Research Resources, Penn State University College of Medicine, 500 University Drive, Hershey, PA, 17033, USA

**Keywords:** Catabolite inactivation, Catabolite repression, Glycolysis, Gluconeogenesis, FBPase, *Saccharomyces cerevisiae*, iTRAQ, MALDI

## Abstract

**Background:**

When glucose is added to *Saccharomyces cerevisiae* grown in non-fermentable carbon sources, genes encoding ribosomal, cell-cycle, and glycolytic proteins are induced. By contrast, genes involved in mitochondrial functions, gluconeogenesis, and the utilization of other carbon sources are repressed. Glucose also causes the activation of the plasma membrane ATPase and the inactivation of gluconeogenic enzymes and mitochondrial enzymes. The goals of this study were to use the iTRAQ-labeling mass spectrometry technique to identify proteins whose relative levels change in response to glucose re-feeding and to correlate changes in protein abundance with changes in transcription and enzymatic activities. We used an experimental condition that causes the degradation of gluconeogenic enzymes when glucose starved cells are replenished with glucose. Identification of these enzymes as being down-regulated by glucose served as an internal control. Furthermore, we sought to identify new proteins that were either up-regulated or down-regulated by glucose.

**Results:**

We have identified new and known proteins that change their relative levels in cells that were transferred from medium containing low glucose to medium containing high glucose. Up-regulated proteins included ribosomal subunits, proteins involved in protein translation, and the plasma membrane ATPase. Down-regulated proteins included small heat shock proteins, mitochondrial proteins, glycolytic enzymes, and gluconeogenic enzymes. Ach1p is involved in acetate metabolism and is also down-regulated by glucose.

**Conclusions:**

We have identified known proteins that have previously been reported to be regulated by glucose as well as new glucose-regulated proteins. Up-regulation of ribosomal proteins and proteins involved in translation may lead to an increase in protein synthesis and in nutrient uptake. Down-regulation of glycolytic enzymes, gluconeogenic enzymes, and mitochondrial proteins may result in changes in glycolysis, gluconeogenesis, and mitochondrial functions. These changes may be beneficial for glucose-starved cells to adapt to the addition of glucose.

## Background

*Saccharomyces cerevisiae* is an excellent model system to study cellular responses to a variety of environmental changes such as oxidative stress, temperature, aerobic versus anaerobic conditions, and the availability of carbon or nitrogen sources [1-15]. Yeast can obtain energy through fermentation of various sugars including glucose, fructose, sucrose, galactose, melibiose, and maltose [1-6]. Yeast can also obtain energy through the utilization of non-fermentable carbon sources such as glycerol, pyruvate, acetate, and lactate [1-6,16].

**Figure 1:**
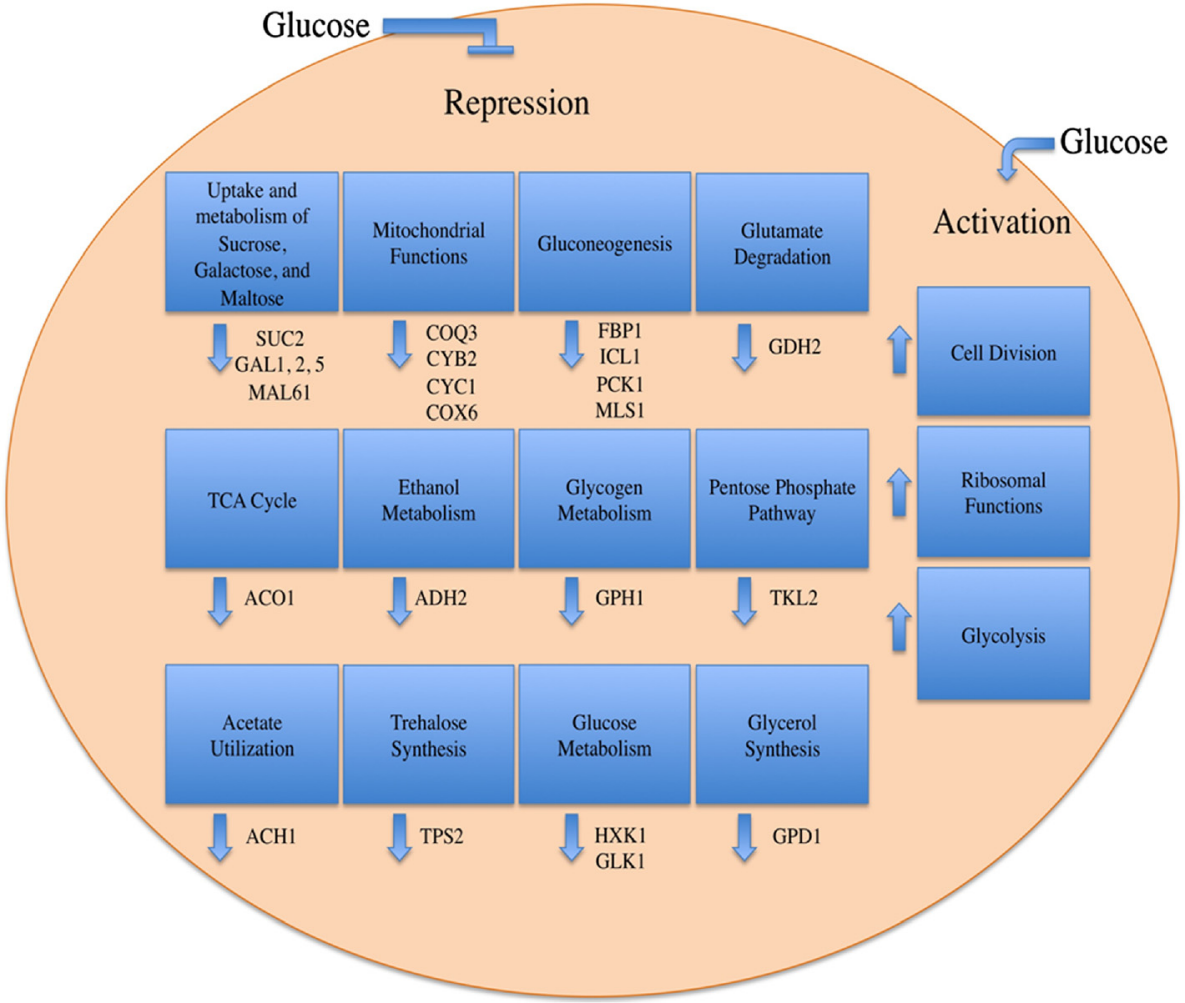
**Diagram showing genes that are repressed by glucose and genes that are induced by glucose.** Glucose-repressible genes include genes involved in the uptake and metabolism of sucrose, galactose, and maltose. Glucose also represses genes involved in mitochondrial functions, gluconeogenesis, glutamate degradation, ethanol metabolism, glycogen metabolism, the pentose pathway, acetate metabolism, trehalose synthesis, and glycerol synthesis. A number of genes involved in cell division, ribosomal functions, and in glycolysis are induced by glucose.

Growth of yeast in non-fermentable carbon sources induces metabolic pathways required for the utilization of these carbon sources [6,16], and the addition of glucose to cells previously grown in non-fermentable carbon sources results in a rapid change in the transcriptional state of the genome [6,17]. An estimated 40% of genes in yeast alter their expression by more than two-fold within minutes following the addition of glucose to cells grown in a non-fermentable carbon source [17]. In general, glucose increases the expression of genes involved in glycolysis, ribosomal functions, and cell division (Figure 1) [1-6,16-27]. Glucose also represses genes required for mitochondrial functions, genes encoding the gluconeogenic enzymes such as FBP1 (fructose-1,6-bisphosphatase), ICL1 (isocitrate lyase), PCK1 (phosphoenolpyruvate carboxykinase), and MLS1 (malate synthase) [4,6,17,26,28-30], and genes required for metabolism of sugars other than glucose and fructose (Figure 1). The repression of genes by glucose is referred to as “catabolite repression” [4,16,17,28,31-33]. Complex regulatory networks that interconnect and overlap at different levels mediate glucose-induced changes in transcriptional activation or repression. For example, the Ras/PKA pathway, the Gpr/Gpa circuit, the Sch9 pathway, the Rgt network, and the Snf1 network have important roles in transcriptional regulation of glucose-repressible or glucose-inducible genes [4,6,16,25,29,34-37].

The effect of glucose is not restricted to transcriptional activation or repression. Glucose also causes a change in the concentration of mRNA. In the presence of a non-fermentable carbon source such as glycerol, the steady-state levels of mRNA for SDH2 and CYC1 are elevated by a combination of induced transcription in the nucleus and stabilization of the mRNAs in the cytoplasm [38]. The half-life of CYC1 mRNA is reduced in de-repressed cells from 12 min to 2 min in the presence of glucose [34,38]. Likewise, the turnover rates of mRNAs for PCK1 and FBP1 are also accelerated by glucose [4,5,16,34,39,40]. In contrast, the turnover rates of mRNAs for the 40S and 60S ribosomal subunits are reduced in response to high glucose [17,38].

Glucose affects enzymatic activities not only through a change in the rate of transcription, but also through an increase in the rate of degradation of proteins. This is known as “catabolite inactivation” [41-44]. Increased degradation of fructose-1,6-bisphosphatase (Fbp1p), isocitrate lyase (Icl1p), the cytosolic malate dehydrogenase (Mdh2p), and phosphoenolpyruvate carboxykinase (Pck1p) are responsible for catabolite inactivation of these enzymes [40,45-52]. Inactivation and degradation of gluconeogenic enzymes during glucose re-feeding prevents energy futile cycles that could be detrimental to cells. In addition to the inactivation of these gluconeogenic enzymes, glucose also reduces the activities of mitochondrial enzymes including aconitase, cytochrome c oxidase, NADH dehydrogenase, and the mitochondrial ATPase [34,53-55].

In contrast to the inactivation of gluconeogenic enzymes and mitochondrial enzymes, glucose causes the activation of the plasma membrane H^+^-ATPase (Pma1p) [4,56-58]. Pma1p pumps protons out of cells to create a proton gradient needed for the uptake of nutrients into cells. The activation of Pma1p is rapid and is a reversible process. In the presence of glucose, the Km for ATP is reduced leading to an activation of this enzyme [56]. Phosphorylation of Pma1p is critical for the process of activation [4,56-58].

Although analysis of mRNA is a powerful way to understand functional mechanisms of the entire genome, it is not sufficient for full characterization of biological systems. Much evidence has accumulated indicating that mRNA abundance is not always correlated with levels of protein expression, and changes in mRNA abundance coding for a particular protein clearly has no direct bearing on whether or not that protein is activated or inactivated by post-translational modifications, including changes in the rate of degradation. Poor correlation between transcriptome and proteome levels has been observed for proteins involved in glycolysis, gluconeogenesis, purine metabolism, and amino acid metabolism [11,59]. For instance, microarray experiments have identified several mitochondrial genes that are regulated in response to glucose. However, proteome studies indicate that mitochondrial proteins are remarkably constant whether cells are grown in glucose or in non-fermentable carbon sources [59]. Since mRNA levels are not perfect indicators of protein levels, we sought to use comparative proteomics to identify proteins that are up-regulated or down-regulated by glucose and to correlate changes in protein abundance with changes in transcription and enzymatic activities.

Glucose effects on protein expression levels have been described in previous proteomic studies that examined steady state levels of protein expression in cells grown in different carbon sources for a prolonged period of time (summarized in Table 1). Because steady state levels of proteins are determined by protein synthesis, protein degradation, or both, the observed low expression levels of gluconeogenic enzymes in cells grown in glucose are likely to result from low rates of protein synthesis. In contrast, during catabolite inactivation, existing gluconeogenic enzymes are rapidly degraded in response to glucose. In cells that are starved of glucose, gluconeogenic enzymes have half-lives of longer than 100 hours. When glucose is added to glucose-starved cells, half-lives of these enzymes are reduced to 20–40 min [48,52,60,61]. For the degradation of FBPase, cAMP is transiently increased which activates the RAS2/PKA signaling pathway [62-68]. This leads to the phosphorylation of FBPase [62-68] and subsequent degradation of this protein. Therefore, the molecular mechanisms for catabolite inactivation of gluconeogenic enzymes are fundamentally different from low rates of protein synthesis in cells grown in glucose. Given that glucose represses a large number of genes involved in different metabolic pathways, we hypothesize that glucose effects on catabolite inactivation are not restrictive to gluconeogenic enzymes. To identify new proteins that are regulated by glucose, we sought to use an experimental condition to reproduce glucose effects on catabolite inactivation of gluconeogenic enzymes.

**Table 1:** Comparison of previous proteomic studies of protein expression in yeast cells grown in various conditions

**Study**	**Francesca et al.**[10]	**de Groot et al.**[11]	**Usaite et al.**[12]	**Costenoble et al.**[13]	**Kolkman et al.**[14]	**Pham et al.**[15]
**Primary Objectives**	Comparison of steady state protein levels in cells growth in synthetic medium containing 0.5%, 2%, and 20% glucose to 0.8 O.D./ml	Proteomic differences in anaerobic versus aerobic growth	Comparison of steady state levels of proteins in strains deficient in *SNF1/SNF4* involved in glucose repression.	Comparison of steady state levels of proteins in cells grown in glucose, galactose or ethanol.	Comparison of steady state levels of protein expression under chemostat cultures limited for either glucose or ethanol.	Comparison of steady state levels of proteins in cells grown in 120 g/L (normal) to 210 g/L and 300 g/L (high) glucose for 68 hours.
**Analytical Platform employed**	2D-GE; Relative spot volume quantification; MALDI-TOF	Stable-isotope labeling with ^14^ N and ^15^ N in cultures grown in anaerobic versus aerobic conditions; 1D-PAGE; RFLC; nanoflow-LC-ESI-MS/ MS	Stable isotope labeling with ^14^ N and^15^N in wild-type, *Δsnf1*, *Δsnf4,* and *Δsnf1Δsnf4* strains; MudPIT; ESI; LTQ-Orbitrap	Targeted proteomics approach based on selected reaction monitoring (SRM) and proteotypic peptides (PTPs); ion trap MS with nanoelectrospray ion source	2D-GE; Relative spot quantification; MALDI-MS and Nano-ESI-LC-MS/MS	iTRAQ; nano-LC-ESI-MS/MS
**Total number of peptides/proteins identifications/ quantification**	156 protein spots changing significantly; 21 differentially expressed proteins identified by MS analysis	1499 identified; 474 quantified proteins; 249 proteins showed differential expression levels	2388 proteins were relatively quantified; 350 showed differential expression levels	The 228 proteins of the central carbon and amino-acid metabolic network in *S. cerevisiae*	400 protein spots were detected on each 2D gel; 44 spots were relatively quantified and identified	413 proteins were identified from 3 replicates; 237 showed differential expression between conditions
**Relevance to our study**	Gluconeogenic enzymes were not identified.	1. Steady state levels of glycolytic enzymes were higher in cells grown in anaerobic condition.	Steady state levels of gluconeogenic enzymes Mls1p, Icl1p, Mdh2p were higher in the *Δsnf1Δsnf4* strain than the *Δsnf1* strain.	1. Steady state levels of gluconeogenic enzymes were higher in cells grown in ethanol than in cells grown in glucose.	1. Steady state levels of glycolytic enzymes were higher in cultures grown in glucose than cells grown in ethanol.	1. Levels of most glycolytic enzymes were higher in 300 g/L glucose than in normal glucose.
		2. Poor correlation of protein ratios and mRNA ratios for enzymes in glycolysis/ gluconeogenesis.		2. Steady state levels of glycolytic enzymes were higher in cells grown in glucose than in cells grown in ethanol.	2. Gluconeogenic enzymes such as Mls1p, Pck1p, Mdh2p, and Icl1p were expressed only in ethanol. Fbp1p was not identified.	2. Levels of Hsp12p, Hsp26p, and other heat shock proteins were lower in cells grown in high glucose than in cells grown in normal glucose.

The iTRAQ (Isobaric Tags for Relative and Absolute Quantification) technology has been used to quantify relative changes in protein ratios in cells that are grown under various growth conditions [15,69-72]. iTRAQ is a gel-free technique that uses specific reporter molecules to label primary amines of the N-termini of peptides and the side chains of lysine residues, which are then identified and quantitated using mass spectrometry [72]. We used an experimental condition that causes the degradation of gluconeogenic enzymes Fbp1p, Pck1p, Icl1p, and Mdh2p [45-52]. The identification of these enzymes as being down-regulated by glucose served as an internal control. In analysis of additional results from these experiments, we have identified proteins that were up-regulated by glucose. These included Pma1p, subunits of ribosomes, and other proteins involved in protein translation. Up-regulation of these proteins may lead to increased protein synthesis and nutrient uptake. We have also identified proteins that were down-regulated by glucose. These included glycolytic enzymes Hxk1p, Pgi1p, and Pgm2p, gluconeogenic enzymes Fbp1p and Icl1p, mitochondrial protein Atp2p, the major mitochondrial outer membrane protein Om45p, and small heat shock proteins Hsp12p, Hsp26p, and Hsp30p. Down regulation of Pgm2p, Fbp1p, Icl1p, Atp2p, and heat shock proteins in response to glucose is consistent with previous reports. Down-regulation of glycolytic enzymes, gluconeogenic enzymes, and mitochondrial proteins may lead to changes in glycolysis, gluconeogenesis, and mitochondrial functions when cells are transferred from glucose-deficient medium to glucose-rich medium.

## Results

### Proteins identified by iTRAQ

**Figure 2:**
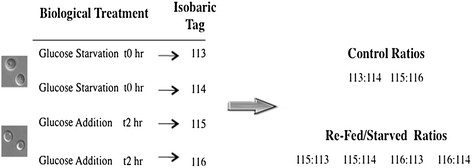
**iTRAQ experimental design.** Duplicates of wild-type cells that were starved of glucose were harvested. Samples were processed, digested with trypsin, and the resulting peptides were labeled with 113 and 114. Duplicates of cells that were glucose-starved and then transferred to medium containing glucose for 2 hours were harvested. Samples were processed and digested with trypsin. The resulting tryptic digested peptides were labeled with 115 and 116. The 113/114 and 115/116 ratios were used as control ratios (replicates in one MS run). The ratios of 115/113, 115/114, 116/113, and 116/114 were used as re-fed/starved ratios.

To identify new proteins that are regulated by glucose, we used an experimental condition that causes the degradation of gluconeogenic enzymes during glucose re-feeding. The experimental designs for these experiments are shown in Figure 2. Briefly, wild-type yeast cells were grown in glucose-deficient media for three days to induce gluconeogenic enzymes. Aliquots of cells were harvested at t = 0 min. The remaining cells were harvested, washed, and re-suspended in media containing fresh glucose for 2 hours; a time period sufficient for degradation of the majority of Fbp1p, Pck1p, Mdh2p, and Icl1p proteins [45,46,48,52]. After cells were harvested, total lysates were obtained and the proteins were digested with trypsin. Resulting tryptic peptide fragments were labeled with the iTRAQ tags, with duplicate t0 cells labeled with 113 and 114 tags, whereas peptides from t2 cells were labeled with 115 and 116 tags. We have identified 591 proteins with an estimated local false discovery rate of less than 0.05. We determined the number of peptides with a confidence interval higher than 95% contributing to the ID of each of these proteins. A total of 153 proteins of these 591 were identified based on a single peptide fragment.

It has been reported previously that more than 50% of the protein IDs based on single peptides could be confirmed as correct [73]. We therefore compared the distribution of biological functions of the 438 proteins that were identified with two or more peptides (Table 2B) to the 153 proteins that were identified with a single peptide (Table 2C). We also compared the distribution of these proteins with the entire genome that contains 6310 proteins (Table 2A). Using the Gene Ontology Slim Mapper program available at the *S. cerevisiae* Genome Database, these proteins were classified into 44 biological processes. Based on the distribution of proteins in biological processes, it appears that we have identified more proteins involved in protein translation, ribosome biogenesis, carbohydrate metabolism, amino acid metabolism, and the generation of metabolites and energy compared to the distribution of the 6310 proteins from the entire genome (Table 2).

**Table 2:** **Cellular functions of proteins identified and gene ontology (GO) annotations from the *saccharomyces *genome database**

	**A**		**B**		**C**	
	**Genome**	**n = 6310**	**Peptides > 1**	**n = 438**	**Peptides = 1**	**n = 153**
**GO term FUNCTION**	**Frequency**	**Percent**	**Frequency**	**Percent**	**Frequency**	**Percent**
biological process unknown	1217	19.3	33	7.5	14	9.2
RNA metabolic process	1210	19.2	64	14.6	20	13.1
transport	1047	16.6	88	20.1	33	21.6
translation	706	11.2	129	29.5	17	11.1
transcription, DNA-dependent	603	9.6	15	3.4	7	4.6
response to stress	594	9.4	61	13.9	16	10.5
protein modification process	583	9.2	22	5.0	11	7.2
cell cycle	527	8.4	18	4.1	6	3.9
ribosome biogenesis	411	6.5	50	11.4	11	7.2
DNA metabolic process	404	6.4	15	3.4	5	3.3
chromosome organization	398	6.3	18	4.1	6	3.9
vesicle-mediated transport	366	5.8	20	4.6	13	8.5
response to chemical stimulus	331	5.2	30	6.8	11	7.2
mitochondrion organization	318	5.0	22	5.0	8	5.2
cellular membrane organization	286	4.5	24	5.5	10	6.5
heterocycle metabolic process	269	4.3	44	10.0	15	9.8
cellular carbohydrate metabolic process	265	4.2	53	12.1	12	7.8
cellular amino acid metabolic process	239	3.8	63	14.4	17	11.1
signaling	235	3.7	8	1.8	6	3.9
cytoskeleton organization	228	3.6	16	3.7	11	7.2
cellular lipid metabolic process	223	3.5	10	2.3	4	2.6
protein complex biogenesis	212	3.4	17	3.9	10	6.5
cellular protein catabolic process	198	3.1	15	3.4	5	3.3
cofactor metabolic process	163	2.6	33	7.5	4	2.6
generation of precursor metabolites and energy	160	2.5	53	12.1	4	2.6
meiosis	157	2.5	7	1.6	2	1.3
cellular homeostasis	150	2.4	20	4.6	7	4.6
chromosome segregation	142	2.3	3	0.7	1	0.7
fungal-type cell wall organization	133	2.1	5	1.1	3	2.0
vacuole organization	130	2.1	12	2.7	3	2.0
sporulation resulting in formation of a cellular spore	128	2.0	10	2.3	3	2.0
conjugation	117	1.9	5	1.1	2	1.3
cytokinesis	110	1.7	4	0.9	3	2.0
transposition	108	1.7	1	0.2	0	0.0
cellular component morphogenesis	96	1.5	3	0.7	3	2.0
protein folding	88	1.4	27	6.2	3	2.0
cellular respiration	88	1.4	21	4.8	2	1.3
cell budding	85	1.3	6	1.4	3	2.0
cellular aromatic compound metabolic process	77	1.2	13	3.0	7	4.6
vesicle organization	77	1.2	2	0.5	3	2.0
peroxisome organization	67	1.1	2	0.5	0	0.0
pseudohyphal growth	66	1.0	5	1.1	0	0.0
vitamin metabolic process	63	1.0	2	0.5	1	0.7
nucleus organization	60	1.0	4	0.9	2	1.3

Peptides used for identification include only those having at least 95% confidence. (A) The distribution of 6310 proteins, (B) 438 proteins identified with more than one peptide and (C) 153 proteins identified with one peptide.

We next sorted these proteins based on their subcellular distributions (Table 3). Some proteins are distributed in multiple locations and have been counted more than once using this program. For the entire genome of 6310 proteins, the largest numbers of proteins (60.4%) are distributed in the cytoplasm. A significant percentage of proteins are also found in the nucleus (33.0%), mitochondria (17.8%), and in unknown locations (12.3%). For the 438 proteins that were identified using more than two peptides, the majority of proteins were found in the cytoplasm (93.2%). It appears that we have identified higher percentages of proteins located in the cytoplasm, mitochondria, ribosomes, and membrane fractions compared to the 6310 proteins from the yeast genome. For the 153 proteins that were identified with one peptide, we did not find proteins located in peroxisomes or in the extracellular region. It is possible that proteins in these compartments are difficult to be digested with trypsin and would therefore be more likely to contain fewer peptides for identification.

**Table 3:** **Subcellular localization of proteins identified and gene ontology (GO) annotations from the *saccharomyces *genome database**

	**A**		**B**		**C**	
	**Genome**	**n = 6310**	**Peptide >1**	**n =438**	**Peptide =1**	**n =153**
**GO term LOCATION**	**Frequency**	**Percent**	**Frequency**	**Percent**	**Frequency**	**Percent**
cytoplasm	3812	60.4	408	93.2	126	82.4
nucleus	2080	33.0	88	20.1	53	34.6
mitochondrion	1126	17.8	137	31.3	28	18.3
unknown	779	12.3	4	0.9	6	3.9
ER	375	5.9	18	4.1	17	11.1
ribosome	355	5.6	102	23.3	17	11.1
plasma membrane	288	4.6	12	2.7	4	2.6
vacuole	221	3.5	20	4.6	2	1.3
cytoskeleton	208	3.3	13	3.0	9	5.9
membrane fraction	208	3.3	52	11.9	7	4.6
Golgi	186	2.9	5	1.1	7	4.6
cytoplasmic membrane-bounded vesicle	107	1.7	5	1.1	6	3.9
cell wall	98	1.6	6	1.4	1	0.7
peroxisome	64	1.0	7	1.6	0	0.0
extracellular region	27	0.4	3	0.7	0	0.0

Peptide used for identification include only those having at least 95% confidence. (A) The distribution of 6310 proteins, (B) 438 proteins identified with more than one peptide and (C) 153 proteins identified with one peptide.

To be accepted as a significant quantitative difference between treatment combinations, we imposed the following criteria: the affected proteins with three of the four iTRAQ ratios significantly higher than 1.0 (p-value less than 0.05) were considered to be up-regulated (Table 4A). Conversely, proteins with three of the four iTRAQ ratios that were significantly lower than 1.0 (p-value less than 0.05) were considered to be down-regulated (Table 5A). Furthermore, the ratios of 113/114 and 115/116 (or 114/113 and 116/115) of these proteins should be close to one (Tables 4B and 5B). Finally, we only included proteins whose identification was based on more than one peptide with a confidence greater than 95%.

**Table 4:** Proteins up-regulated by glucose

**A**											
**Name**	**115:113**	**P**	**115:114**	**P**	**116:113**	**P**	**116:114**	**P**	**Ave**	**SD**	**t-test**
Rps0bp, 40S Ribosomal Subunit	2.1677	0.0324	2.2909	0.0133	1.9588	0.0369	2.0324	0.0152	2.1125	0.1274	0.0006
Rps8bp, 40S Ribosomal Subunit	2.3768	0.0015	3.8726	0.0006	1.7865	0.0080	2.8576	0.0008	2.7234	0.7643	0.0298
Rpl3p, 60S Ribosomal Subunit	1.3428	0.0266	1.5136	0.0005	1.1482	0.2264	1.2942	0.0056	1.3247	0.1305	0.0230
Rpl7bp, 60S Ribosomal Subunit	1.9950	0.0129	1.7380	0.0241	1.8540	0.0121	1.6290	0.0320	1.8040	0.1360	0.0020
Tef2p, Translational Elongation Factor EF-1 alpha	2.4434	0.0134	2.3550	0.0422	2.2491	0.0146	2.1677	0.0254	2.3038	0.1044	0.0002
Tef4p, Translational Elongation Factor eEF1B	2.5586	0.0441	1.9588	0.0281	2.5119	0.0528	1.9231	0.0275	2.2381	0.2979	0.0055
Lia1p, Deoxyhypusine Hydroxylase	2.9107	0.0115	3.3113	0.0081	2.6546	0.0169	3.0479	0.0116	2.9811	0.2372	0.0007
Pma1p, Plasma Membrane H+-ATPase	1.4859	0.0112	2.0701	0.0020	1.5276	0.0224	2.1281	0.0032	1.8029	0.2972	0.0184
**B**											
**Accession #**	**Name**					**Peptides (95%)**	**113:114**	**P**	**115:116**	**P**	**t-test**
gi|6323077	Rps0bp, 40S Ribosomal Subunit					12	1.0471	0.5788	1.0864	0.9352	0.1823
gi|6320949	Rps8bp, 40S Ribosomal Subunit					14	1.6596	0.2979	1.3428	0.4164	0.1949
gi|6324637	Rpl3p, 60S Ribosomal Subunit					23	1.1272	0.0651	1.1695	0.2455	0.0902
gi|6325058	Rpl7bp, 60S Ribosomal Subunit					14	0.8318	0.6646	1.0186	0.8621	0.5701
gi|99031872	Tef2p, Translational Elongation Factor EF-1 alpha					63	0.9727	0.8135	1.0666	0.9691	0.7477
gi|6322769	Tef4p, Translational Elongation Factor eEF1B					10	0.7244	0.7899	1.0000	0.9030	0.5000
gi|6322531	Lia1p, Deoxyhypusine Hydroxylase					4	1.1588	0.7297	1.0765	0.7288	0.2142
gi|6321430	Pma1p, Plasma Membrane H+-ATPase					12	1.4454	0.5425	0.9462	0.8061	0.5765

(A) Proteins that increased their relative levels in response to glucose re-feeding. Average ratios (Avg) and standard deviations (SD) are given, along with the p-values (P) calculated for each protein's ratio by ProteinPilot^TM^ 4.0 based on the ratios of each identified peptide. A one-sample Student's t-test was used to confirm that each protein's set of ratios for all four comparisons was significantly different from 1 and all p-values are <0.05.

(B) Ratios of 113:114; starved:starved and 115:116; re-fed:re-fed with the number of distinct peptides having at least 95% confidence and a p-value (P) greater than 0.05 calculated for each protein's ratio by ProteinPilot^TM^ 4.0 based on the ratios of each identified peptide. A one-sample Student's t-test was used to confirm that each protein's set of ratios for both comparisons was not significantly different from 1 and all p-values are >0.05.

**Table 5:** Proteins down-regulated by glucose

**A**											
**Name**	**115:113**	**P**	**115:114**	**P**	**116:113**	**P**	**116:114**	**P**	**Ave**	**SD**	**t-test**
Hxk1p, Hexokinase A	0.3908	0.0002	0.4529	0.0025	0.5200	0.0015	0.5970	0.0181	0.4902	0.0768	0.0014
Pgi1p, Phosphoglucose Isomerase	0.6792	0.0267	0.4529	0.0042	0.7178	0.2011	0.4613	0.0416	0.5778	0.1215	0.0092
Pgm2p, Phosphoglucomutase	0.4875	0.0137	0.4246	0.0068	0.6918	0.0936	0.6026	0.0476	0.5516	0.1031	0.0048
Fbp1p, Fructose-1,6-Bisphosphatase	0.1528	0.0001	0.1259	0.0001	0.0731	0.0001	0.0625	0.0001	0.1036	0.0372	0.0001
Icl1p, Isocitrate Lyase	0.3281	0.0001	0.2630	0.0001	0.2630	0.0001	0.2128	0.0001	0.2667	0.0409	0.0001
Mls1p, Malate Synthase	0.5395	0.0062	0.4285	0.0019	0.7656	0.05704	0.5916	0.0175	0.5813	0.1216	0.0094
Ach1p, Acetyl CoA Hydrolase	0.4831	0.0021	0.5152	0.0036	0.6546	0.0213	0.6855	0.0513	0.5846	0.0869	0.0037
Atp2p, Mitochondrial ATP Synthase Subunit Beta	0.4966	0.0059	0.2938	0.0053	0.6252	0.0219	0.3597	0.0200	0.4438	0.1277	0.0048
Om45p, Mitochondrial Outer Membrane Protein	0.4613	0.0045	0.5702	0.0144	0.4656	0.0020	0.5649	0.0064	0.5155	0.0521	0.0005
Hsp12p, Heat Shock Protein	0.3565	0.0091	0.3251	0.0945	0.2630	0.0020	0.2466	0.0126	0.2978	0.0448	0.0001
Hsp26p, Heat Shock Protein	0.5445	0.0130	0.5861	0.0162	0.3698	0.0013	0.4018	0.0016	0.4756	0.0916	0.0022
Hsp30p, Heat Shock Protein	0.3981	0.0922	0.3221	0.0446	0.2312	0.0393	0.1905	0.0175	0.2479	0.0550	0.0006
**B**											
**Accession #**	**Name**					**Peptides (95%)**	**113:114**	**P**	**115:116**	**P**	**t-test**
gi|6321184	Hxk1p, Hexokinase A					20	1.3804	0.7068	0.9462	0.9299	0.5894
gi|6319673	Pgi1p, Phosphoglucose Isomerase					26	0.6368	0.3942	0.9204	0.2883	0.3626
gi|817863	Pgm2p, Phosphoglucomutase					18	0.8241	0.7017	0.6730	0.2974	0.1858
gi|6323409	Fbp1p, Fructose-1,6-Bisphosphatase					7	0.8166	0.1819	1.9953	0.1888	0.6160
gi|6320908	Icl1p, Isocitrate Lyase					14	0.7870	0.2416	1.2589	0.1990	0.9383
gi|6324212	Mls1p, Malate Synthase					13	0.7586	0.5169	0.7112	0.2358	0.0568
gi|6319456	Ach1p, Acetyl CoA Hydrolase					16	1.0375	0.6989	0.7311	0.1846	0.5882
gi|84028178	Atp2p, Mitochondrial ATP Synthase Subunit Beta					20	0.6081	0.9613	0.8472	0.4900	0.2633
gi|730224	Om45p, Mitochondrial Outer Membrane Protein					17	1.2023	0.5644	0.9908	0.6866	0.5289
gi|836740	Hsp12p, Heat Shock Protein					35	0.9462	0.2305	1.3428	0.4280	0.5991
gi|6319546	Hsp26p, Heat Shock Protein					41	1.0965	0.8963	1.4859	0.1689	0.3752
gi|6319869	Hsp30p, Heat Shock Protein					6	0.7943	0.4318	1.7701	0.5325	0.6662

(A) Proteins that decreased their relative levels in response to glucose re-feeding. Average ratios (Avg) and standard deviations (SD) are given, along with the p-values (P) calculated for each protein's ratio by ProteinPilot^TM^ 4.0 based on the ratios of each identified peptide. A one-sample Student's t-test was used to confirm that each protein's set of ratios for all four comparisons was significantly different from 1 and all p-values are <0.01.

(B) Ratios of 113:114; starved:starved and 115:116; re-fed:re-fed with the number of distinct peptides having at least 95% confidence and a p-value (P) greater than 0.05 calculated for each protein's ratio by ProteinPilot^TM^ 4.0 based on the ratios of each identified peptide. A one-sample Student's t-test was used to confirm that each protein's set of ratios for both comparisons was not significantly different from 1 and all p-values are >0.05.

### Proteins Up-Regulated by Glucose

Previous work has shown that the presence of high glucose induces the transcription of more than 90% of the ribosomal protein genes 2–4 fold within 30 minutes [17]. In one proteomic study, 22 ribosomal subunits were identified and 9 subunits increased their relative levels in response to 300 g/L glucose [15]. Because we used a different experimental condition to study glucose effects, we examined whether or not ribosomal subunits increase their relative levels when glucose starved cells were transferred to medium containing fresh glucose for 2 hours.

We have identified subunits of ribosomes that were up-regulated by glucose (Table 4). Rps0bp and Rps8ap are components of the small (40S) ribosomal subunit, whereas Rpl3p and Rpl7bp are subunits of the 60S ribosomes [19,74,75].

We have also identified three proteins involved in different aspects of protein translation that were up-regulated. Tef2p is the translational elongation factor EF-1 alpha and functions in the binding reaction of aminoacyl-tRNA to ribosomes [76]. Tef4p is the gamma subunit of the translational elongation factor eEF1B. Tef4p stimulates the binding of aminoacyl-tRNA to ribosomes by releasing eEF1A from the ribosomal complex [77]. Lia1p is deoxyhypusine hydroxylase that catalyzes the formation of hypusine required for the modification of eIF5A [78]. Tef2p, Tef4p, and Lia1p have not been previously reported to be up-regulated by glucose.

The plasma membrane ATPase was identified as being up-regulated and was included in this group. Pma1p pumps protons out of the cell and is the major regulator of cytoplasmic pH and plasma membrane potential [4,79,80]. This protein is highly regulated by glucose both transcriptionally and post-translationally, as glucose not only induces PMA1 gene expression but also activates ATPase activity [4,79,80]. In the current study, we found that relative levels of Pma1p were higher following the addition of glucose. Pma1p is an abundant protein and is negatively regulated by Hsp30p [81]. Interestingly, we also observed a down-regulation of Hsp30p in this study (see Table 5).

### Proteins Down-Regulated by Glucose

The presence of high glucose reduces the transcription of genes involved in gluconeogenesis, glyoxylate cycle, and the TCA cycle [16,17]. Furthermore, glucose also causes the degradation of gluconeogenic enzymes. Although a recent mitochondrial proteomic study has indicated that mitochondrial proteins are remarkably constant [59], earlier studies have shown that the mitochondrial F1 subunits decrease their levels in glucose–repressed cells [82].

In our current study, we have identified 12 proteins that were down-regulated by glucose (Table 5A). These proteins are involved in gluconeogenesis and mitochondrial functions. Small heat shock proteins were also identified in this group. Down-regulation of proteins in these functional groups correlates with the observation that genes in these same categories are repressed by glucose.

For glycolytic enzymes that were down-regulated by glucose, Hxk1p is involved in the phosphorylation of glucose at the C6 position in the first irreversible step in glucose metabolism [83,84]. Pgi1p (phosphoglucose isomerase) catalyzes the interconversion of glucose-6-phosphate and fructose-6-phosphate [85]. Our iTRAQ data indicate that relative levels of Hxk1p and Pgi1p were reduced following the addition of glucose. Pgm2p (Gal5p) is the major isoform of phosphoglucomutase that catalyzes the interconversion of glucose-1-phosphate to glucose-6-phosphate. As such, Pgm2p is involved in glycolysis, the pentose phosphate pathway, and the metabolism of glycogen, galactose, and trehalose [86]. Glucose not only represses transcription of PGM2 but also reduces activity of Pgm2p [17,86,87]. Consistent with the down-regulation of the PGM2 gene and its activity, the addition of glucose to glucose-starved cells caused a decrease in the abundance of Pgm2p (Table 5).

As mentioned, we used the known glucose-induced degradation of four gluconeogenic enzymes as our internal control. Indeed, we detected a very dramatic decline in protein levels for Fbp1p and Icl1p. Fbp1p is a key enzyme in the irreversible steps of gluconeogenesis. Fbp1p converts fructose-1,6-bisphosphate to fructose-6-bisphosphate, and the gene coding for Fbp1p is repressed by glucose [4,34]. Furthermore, this protein is also degraded in response to glucose [45-51]. Consistent with these observations, levels of Fbp1p were reduced following the addition of glucose. Another gluconeogenic enzyme, Mdh2p, was identified in this study and showed a decrease in abundance following the addition of glucose. However, the p-value was higher than 0.05. As such, this protein was not included in Table 5. Pck1p is another key enzyme in the irreversible step of gluconeogenesis. It catalyzes the formation of phosphoenolpyruvate from oxaloacetate. Pck1p was also identified in this study and showed a dramatic decrease in levels in response to glucose. However, the ratios of 115/116 or 116/115 were scattered. Hence, this protein was not included in Table 5. Our stringent criteria may result in underestimation of the number of proteins that alter their abundance in response to glucose re-feeding.

Two additional enzymes in gluconeogenesis/glyoxylate pathway, isocitrate lyase (Icl1p) and malate synthase (Mls1p), were also down-regulated by glucose (Table 5). Icl1p catalyzes the formation of succinate and glyoxylate from isocitrate [88], whereas Mls1p catalyzes the formation of malate from glyoxylate in the glyoxylate cycle [89]. Levels of these proteins were reduced following the addition of glucose.

ACH1 encodes CoA transferase that hydrolyses acetyl-CoA and transfers CoASH from succinyl-CoA to acetate. Glucose causes the repression of the ACH1 gene and a reduction in Ach1p activity [30]. We showed that levels of Ach1p were also reduced when glucose was added to glucose-starved cells (Table 5).

It has been documented that glucose suppresses genes encoding mitochondrial proteins and reduces the activity of several mitochondrial enzymes such as NADH dehydrogenase, aconitase, cytochrome c oxidase, and the mitochondrial ATPase [34,82]. The presence of high glucose not only reduces ATPase activity but also decreases levels of the F1 subunits [82]. F1 consists of Atp1p and Atp2p and is the catalytic subunits of the ATPase [90-92]. Our data showed that levels of Atp2p were also reduced when glucose was added to glucose-starved cells. Another mitochondrial protein Om45p was also down-regulated by glucose. Om45p is a major constituent of the mitochondrial outer membrane, however, the function of Om45p is currently unknown.

We have also identified three heat shock proteins that were down-regulated. Hsp12p is a small heat shock protein localized to the plasma membrane [93,94]. Hsp26p is a heat shock protein with chaperone activity [95]. Hsp30p is a stress-responsive protein localized to the plasma membrane. Hsp30p is reported to negatively regulate Pma1p [81]. Down-regulation of Hsp30p may relieve the inhibitory effects of Hsp30p on Pma1p. Interestingly, many of these proteins such as Hxk1p, Mls1p, Om45p, Hsp26p, and Ach1p were up-regulated when cells were grown in glucose-limited conditions [9]. Here, we show that when glucose-starved cells were transferred to medium containing fresh glucose, levels of these proteins were reduced. Therefore, we suggest that these proteins are highly regulated by the availability of glucose.

### Expression of Subunits in Protein Complexes

Because many proteins do not function on their own but as part of larger protein complexes, we next examined how subunits of some well-described protein complexes are regulated following the regime of glucose depletion and re-addition (Table 6). Phosphofructokinase (PFK) is a key enzyme in glycolysis and catalyzes the formation of fructose-1,6-bisphosphate from fructose-6-phosphate and ATP. PFK is a hetero-oligomeric enzyme composed of four alpha (Pfk1p) and four beta subunits (Pfk2p) [96]. Glucose not only induces the transcription of the PFK1 and PFK2 genes but also stimulates the activity of the PFK enzyme [17,27]. Therefore, we examined whether or not up-regulation of PFK activity correlates with an increase in protein expression. We have identified both Pfk1p and Pfk2p in our study. However, levels of these proteins did not change significantly before or after the addition of glucose for 2 hours (Table 6).

**Table 6:** Protein complexes and the relative quantification of subunits

**Accession #**	**Name**	**Peptides (95%)**	**113:114**	**115:116**	**115:113**	**116:113**	**115:114**	**116:114**
**Phosphofructokinase (2/2)**			**Control**	**Control**				
gi|6321679	Pfk1p	18	0.9908	0.9817	0.9817	0.9908	0.9817	0.9908
gi|172140	Pfk2p	16	0.9727	0.9727	0.9908	1.0093	0.9727	0.9908
**Vacualar H + Atpase (6/15)**			**Control**	**Control**				
gi|6320016	Tfp1p	21	0.9376	0.9550	0.9550	0.9908	0.9036	0.9376
gi|6324844	Vph1p	3	0.9462	1.0000	1.0093	1.0093	0.9638	0.9550
gi|6319603	Vma2p	10	0.9550	0.9908	0.9727	0.9727	0.9376	0.9376
gi|6324907	Vma4p	3	0.8954	0.8872	0.9727	1.0864	0.8790	0.9817
gi|6322770	Vma5p	1	0.9376	1.0568	1.2359	1.1482	1.1588	1.0864
gi|6325293	Vma13p	2	0.8630	1.5136	1.5560	1.0186	1.3552	0.8872
**F0/F1 ATP synthase (11/17)**			**Control**	**Control**				
gi|56404985	Atp1p	26	0.8790	1.1066	0.8017	0.7244	**0.7178**	**0.6546**
gi|84028178	Atp2p	20	0.6081	0.8472	**0.4966**	**0.6252**	**0.2938**	**0.3597**
gi|6319513	Atp3p	4	1.0471	0.6368	**0.4742**	0.7379	**0.5012**	0.7727
gi|6325179	Atp4p	3	1.0186	1.0186	0.9638	0.9376	0.9908	0.9638
gi|849218	Atp5p	5	1.0000	1.0471	0.8017	0.7516	0.8017	0.7586
gi|6322836	Atp7p	2	0.9376	0.9462	0.9120	0.9462	0.8630	0.8954
gi|6323326	Atp14p	2	1.0765	1.0471	0.9462	0.8872	1.0280	0.9638
gi|6324984	Atp15p	1	0.7379	1.0375	1.0568	1.0093	0.7943	0.7586
gi|849198	Atp17p	2	0.9817	0.8241	0.7727	0.9290	0.7656	0.9204
gi|6324495	Atp19p	1	0.8091	0.9908	1.0965	1.0965	0.9036	0.8954
gi|6320529	Tim11p	4	0.8872	0.9376	0.8630	0.9120	0.7727	0.8166
**Small 40S Ribosomal Subunit (25/32)**			**Control**	**Control**				
gi|6323077	Rps0bp	12	1.0471	1.0864	**2.1677**	**1.9588**	**2.2909**	**2.0324**
gi|665976	Rps1ap	18	1.3062	1.2246	0.8551	0.6918	1.1272	0.9120
gi|6321315	Rps2p	9	1.0864	1.1695	1.0965	0.9290	1.2023	1.0280
gi|6322605	Rps4ap	20	1.1272	1.1482	1.6144	1.3804	**1.8365**	**1.5704**
gi|895891	Rps5p	11	0.8166	1.2706	1.7378	1.3428	1.4191	1.1169
gi|6325167	Rps6ap	8	0.8630	1.3305	**1.9231**	1.4723	1.7701	1.3183
gi|758292	Rps7bp	6	1.1066	1.0765	1.1169	1.0280	1.2359	1.1376
gi|6320949	Rps8bp	14	1.6596	1.3428	**2.3768**	**1.7865**	**3.8726**	**2.8576**
gi|6319666	Rps9bp	14	1.0186	1.1912	**1.8707**	1.5417	1.9055	1.5704
gi|899490	Rps12p	4	0.8954	0.9550	1.0186	1.0568	0.9204	0.9550
gi|798915	Rps13p	4	1.0186	1.0186	1.0568	1.0280	1.0864	1.0568
gi|730453	Rps14bp	6	1.0280	1.1588	1.1482	0.9817	1.1912	1.0186
gi|6324533	Rps15p	5	1.0765	1.0568	1.0093	0.9550	1.0965	1.0280
gi|9755341	Rps16ap	8	1.0375	1.1376	1.2823	1.1272	1.3552	1.1803
gi|642297	Rps18ap	13	0.8790	1.0375	**1.5849**	**1.4454**	1.4322	1.3305
gi|6324451	Rps19ap	8	1.0093	1.0965	1.0568	0.9550	1.0765	0.9817
gi|730687	Rps20p	5	1.0765	1.0666	1.1376	1.0568	1.2359	1.1482
gi|84028229	Rps21ap	6	0.9727	1.2589	2.2491	1.7539	2.2080	1.7539
gi|6325389	Rps23bp	6	0.9550	1.0280	1.3183	1.2706	1.2706	1.2134
gi|730648	Rps24ap	13	0.8954	1.2942	**1.7378**	1.3305	**1.5704**	1.2023
gi|83288131	Rps25bp	2	1.0765	1.1376	1.1376	0.9908	1.2359	1.0765
gi|730459	Rps26bp	4	1.0375	0.9908	1.1066	1.1066	1.1588	1.1482
gi|730460	Rps27bp	5	0.8954	1.0765	1.2134	1.1169	1.0965	1.0093
gi|85695430	Rps28bp	3	1.0375	0.8872	1.0186	1.1376	1.0765	1.2023
gi|730461	Rps29ap	8	0.9204	0.9036	1.0568	1.1588	0.9817	1.0765
gi|6323196	Rps31p	12	1.3428	0.8017	0.8872	1.1272	1.2474	1.4859
**Large 60S Ribosomal Subunit (38/42)**			**Control**	**Control**				
gi|732951	Rpl1bp	6	1.0280	0.9204	1.0965	1.1803	1.1376	1.2134
gi|730569	Rpl2bp	13	1.4859	1.3062	1.1066	0.8630	1.7865	1.3552
gi|6324637	Rpl3p	23	1.1272	1.1695	**1.3428**	1.1482	**1.5136**	**1.2942**
gi|6325126	Rpl5p	13	1.1482	1.0765	1.9953	1.8365	2.2909	2.0701
gi|6325058	Rpl7bp	14	0.8318	1.0186	**1.9953**	**1.8535**	**1.7378**	**1.6293**
gi|6322984	Rpl8bp	16	1.0965	1.1695	1.3804	1.1695	**1.5136**	1.2823
gi|6321291	Rpl9ap	9	1.2134	1.1376	1.3305	1.1588	**1.6144**	1.4060
gi|747904	Rpl10p	13	1.1588	1.2246	**1.7219**	1.3677	**1.9588**	1.5704
gi|914973	Rpl11ap	3	1.4588	0.9290	1.3677	1.4454	2.0324	1.9953
gi|730531	Rpl13bp	12	1.3804	1.2823	**1.9055**	1.5136	**2.5119**	2.0324
gi|730454	Rpl14bp	7	1.0093	1.000	1.0965	1.0864	1.1169	1.1066
gi|927686	Rpl15ap	5	0.9908	0.9908	1.1376	1.1376	1.1376	1.1376
gi|791117	Rpl16bp	9	0.9376	0.7178	1.2474	1.6144	1.1272	1.5417
gi|6322668	Rpl17ap	6	0.5916	1.1169	2.0512	1.7539	1.2589	1.0864
gi|6324452	Rpl18ap	8	0.5495	1.8030	**2.9648**	1.7701	1.7865	0.9817
gi|6319559	Rpl19ap	7	1.5996	1.1169	1.8707	1.5996	**2.8840**	**2.4660**
gi|940843	Rpl20ap	21	0.7178	1.2246	**1.8365**	1.4723	1.3428	1.0864
gi|6319668	Rpl21ap	6	1.0280	1.0471	1.0666	1.0093	1.0965	1.0375
gi|662127	Rpl22ap	2	0.9290	1.1376	1.2823	1.1169	1.2023	1.0471
gi|6320963	Rpl23bp	4	0.9727	1.0093	1.1482	1.1169	1.1272	1.0965
gi|6321407	Rpl24ap	4	0.9727	1.3183	2.2699	1.6749	2.1478	1.6444
gi|6324445	Rpl25p	7	1.0375	1.2246	1.2706	1.0280	1.3305	1.0765
gi|6323376	Rpl26ap	9	1.0765	1.0000	1.0093	1.0000	1.0965	1.0765
gi|927763	Rpl27bp	13	1.4997	1.2359	1.2023	0.9550	**1.8197**	1.4454
gi|6321335	Rpl28p	7	0.7943	1.2589	2.4660	1.8880	2.0512	1.5276
gi|45270834	Rpl29p	2	0.6026	1.0093	1.2246	1.2134	0.7447	0.7311
gi|6435679	Rpl30p	4	0.8954	1.2823	1.7539	1.3428	1.5704	1.2134
gi|6320128	Rpl31ap	8	0.9908	1.0864	1.1376	1.0471	1.1376	1.0375
gi|6319378	Rpl32p	6	0.6668	1.2589	1.9409	1.5417	1.3062	1.0280
gi|6325114	Rpl33ap	2	0.9727	0.9817	1.0471	1.0568	1.0186	1.0375
gi|9755331	Rpl34ap	1	1.0666	1.1169	1.0186	0.9036	1.1066	0.9817
gi|927770	Rpl35bp	3	0.8395	1.0864	1.1912	1.0864	1.0093	0.9204
gi|6325006	Rpl36bp	4	1.0000	0.9727	0.9908	1.0093	1.0093	1.0186
gi|6323214	Rpl37bp	3	0.9550	1.0471	1.0093	0.9638	0.9727	0.9204
gi|6322272	Rpl39p	1	0.8395	1.0864	1.4454	1.3305	1.2246	1.1169
gi|6322947	Rpl40bp	9	1.0280	1.2134	1.0965	0.8954	1.1482	0.9376
gi|6681849	Rpl42ap	3	1.5136	1.5560	1.556	0.9638	2.3988	1.5276
gi|805027	Rpl43bp	5	1.1588	1.0965	1.5136	1.3804	1.7701	1.6144

Ratios that are significantly different from 1.0 (p < 0.05) **in bold**. 113:114; starved:starved and 115:116; re-fed/re-fed (control ratios). 115:113, 116:113, 115:114, 116:114; re-fed:starved.

The plasma membrane ATPase is a large protein required for acidification of the cell and is up-regulated by glucose (see Table 4). The vacuole ATPase is responsible for the acidification of the vacuole and is a multi-subunit complex consisting of the V0 and V1 subunits [97,98]. Glucose regulates the activity of the vacuolar ATPase in a different way. In the presence of low glucose, V0 and V1 are disassembled and the V1 subunit is distributed in the cytoplasm [97,98]. The addition of glucose to glucose-depleted cells does not change levels of the V-ATPase subunits but causes the V0 and V1 subunits to assemble into a functional ATPase [97,98]. We have identified 6 out of the 15 subunits of the V-ATPase subunits, and levels of these proteins did not change significantly following the addition of glucose to glucose-starved cells. Our results are consistent with the notion that glucose regulates the function of the V-ATPase primarily through the assembly of the V0 and V1 subunits.

Next, we sought to examine how subunits of the mitochondrial ATPase were regulated by glucose. The mitochondrial F0/F1 ATP synthase is a large, evolutionarily conserved complex of enzymes required for ATP synthesis [90,91]. This complex of enzymes consists of a membrane-bound F0, a soluble F1 component, a central and a peripheral stator [90,91]. Interestingly, the assembly of the F1 subunit is independent of the F0 subunit [90,91]. We have identified 11 of 17 subunits of F0/F1 ATP synthase. Levels of Atp1p, Atp2p, and Atp3p were reduced following the addition of glucose. However, most of the ATP synthase subunits did not change their expression levels. Therefore, not all the ATP synthase subunits decreased their levels to the same extent when glucose was added to glucose-starved cells.

The presence of glucose increases mRNAs 2–4 fold for more than 90% of the genes encoding ribosomal proteins [17]. Therefore, we examined whether or not glucose causes similar changes in the abundance of ribosomal subunits. Ribosomes are highly conserved large ribonucleoprotein (RNP) particles consisting of a small 40S subunit and a large 60S subunit required for protein synthesis [17,19,74,75]. The 40S subunit has 32 proteins and the 60S subunit contains 42 proteins [19,74,75]. We have identified 25 subunits of the 40S ribosome and 38 subunits of the 60S ribosome (Table 6). The subunits that showed significant changes in abundance are highlighted. For the 40S ribosomal subunit, fewer than 6 subunits increased their levels, while 19 did not show significant changes. For the 60S ribosomal subunits, fewer than 10 subunits increased their abundance but 28 subunits did not change their levels. Therefore, less than 30% of the ribosomal subunits showed an increase in their abundance in response to glucose under our conditions.

**Figure 3:**
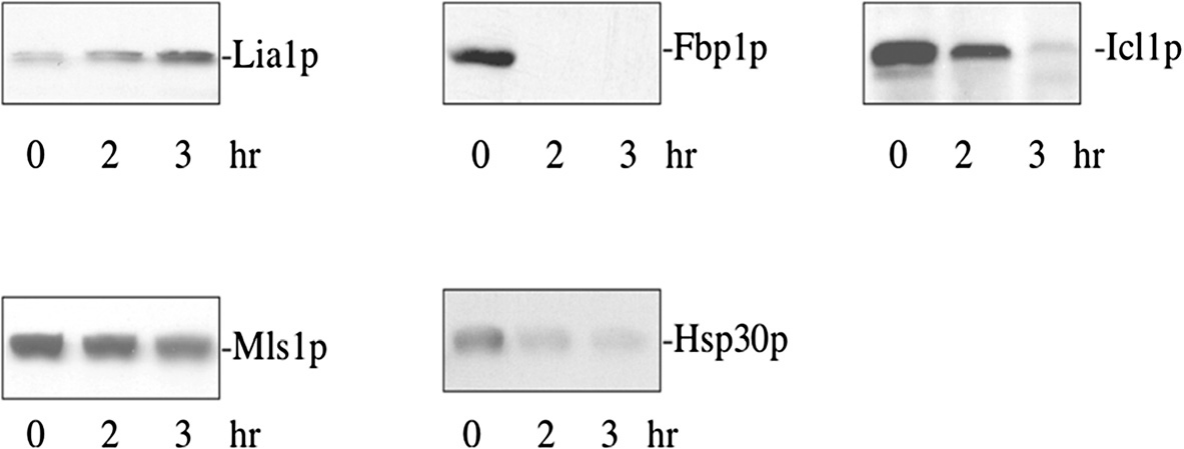
**Lia1p is up-regulated by glucose, whereas Fbp1p, Icl1p, Mls1p, and Hsp30p are down-regulated by glucose.** Cells expressing Lia1p-GFP, Fbp1p-GFP, Icl1p-GFP, Mls1p-GFP, and Hsp30p-GFP were starved of glucose for 3 days and transferred to medium containing glucose for 0, 2, and 3 hours. Levels of proteins were examined by Western blotting using anti-GFP antibodies.

To validate our proteomic data, we examined changes in levels of proteins in response to glucose. Cells expressing Lia1p-GFP, Fbp1p-GFP, Icl1p-GFP, Mls1p-GFP, and Hsp30p-GFP were starved of glucose and then transferred to medium containing high glucose for 0, 2, and 3 hours. Levels of these proteins were then examined by Western blotting with anti-GFP antibodies (Figure 3). In response to glucose, levels of Lia1p-GFP increased. In contrast, levels of Fbp1p-GFP, Icl1p-GFP, Mls1p-GFP, and Hsp30p-GFP were reduced when glucose was added to glucose-starved cells. Therefore, these results confirmed our proteomic data that Lia1p is up-regulated by glucose, whereas Fbp1p, Icl1p, Mls1p, and Hsp30p are down-regulated by glucose.

## Discussion

**Figure 4:**
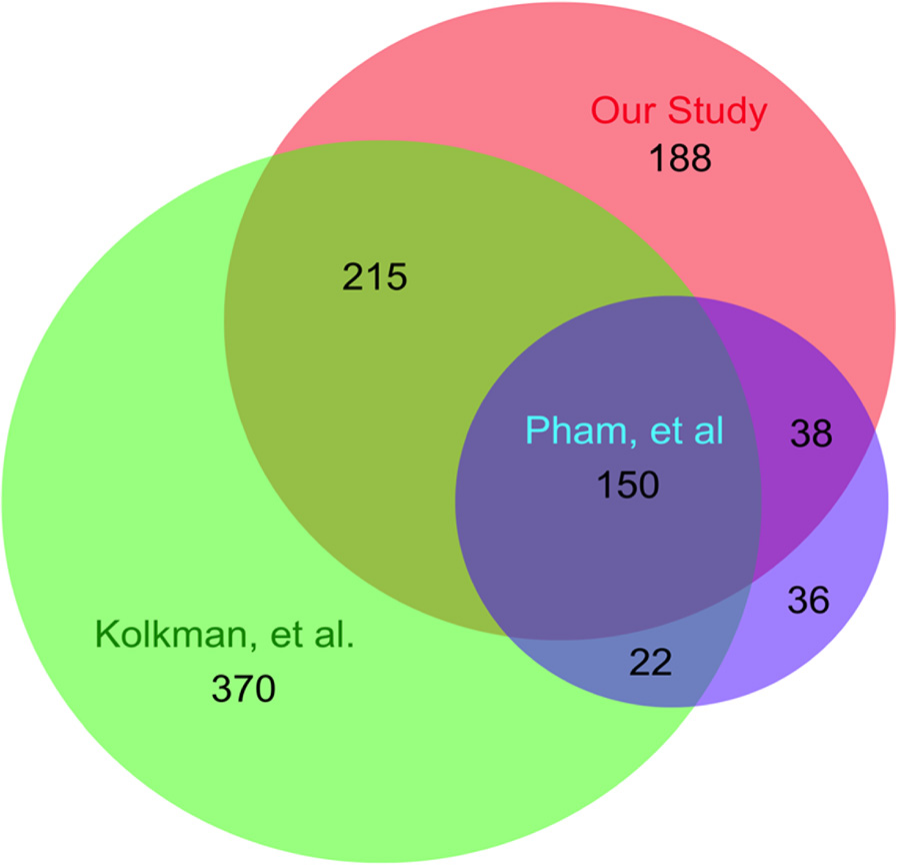
**Comparison of our study with previous proteomic studies.** Overlap in identified proteins from our study and in previous studies reported by Pham et al [15] and by Kolkman et al [9].

In this paper, we report the identification of 591 proteins from yeast cells grown in glucose-deficient medium and transferred to glucose-rich medium for 2 hours using the iTRAQ and MALDI techniques. A previous study by Kolkman et al has identified 928 proteins that were expressed in carbon and nitrogen limitations and 759 proteins were quantified using the SILAC (stable isotope labeling with amino acids in cell culture) and ESI (electrospray ionization) techniques [9]. In another study by Pham et al using the iTRAQ and ESI techniques, 451 proteins were identified and 246 were quantified [15]. When we compared the overlap of proteins identified in these three studies, 150 proteins were present in all three studies (Figure 4). 365 proteins overlapped between our study and the study by Kolkman et al. and 188 proteins overlapped between our study and the study by Pham et al. Furthermore, 188 proteins were unique to our study, whereas 370 proteins were unique to the study by Kolkman et al. and 36 proteins were unique to the study by Pham et al. Given that each study identified unique sets of proteins, these methods appear to complement each other. Again, each of these studies used different experimental conditions. Hxk1p, Mls1p, Ach1p, Om45p, and Hsp26 were up-regulated under carbon limitation in the study by Kolkman et al. (Table 7). Hxk1p, Pgi1p, and Pgm2p were up-regulated, whereas Tef2p, Hsp12p, and Hsp26p were down-regulated in high glucose (300 g/L) (Table 7) in the study by Pham et al.

**Table 7:** **Proteins that were up-regulated and down-regulated by glucose in our study and the comparison with the studies by Kolkman et al.**[9]**and Pham et al**. [15]

**Function**	**Up-regulated in our study**	**Kolkman et al.**[9]	**Pham et al.**[15]
Ribosome	Rps0bp, 40S Ribosomal Subunit	identified	not identified
Ribosome	Rps8bp, 40S Ribosomal Subunit	identified	not identified
Ribosome	Rpl3p, 60S Ribosomal Subunit	identified	not identified
Ribosome	Rpl7bp, 60S Ribosomal Subunit	not identified	not identified
Translation	Tef2p, Translational Elongation Factor EF-1 alpha	not identified	**Down-regulated in high glucose**
Translation	Tef4p, Translational Elongation Factor eEF1B	identified	not identified
Translation	Lia1p, Deoxyhypusine Hydroxylase	identified	not identified
Nutrient Uptake	Pma1p, Plasma Membrane H + −ATPase	**Down-regulated under carbon limitation**	not identified
	**Down-regulated in our study**		
Glycolysis	Hxk1p, Hexokinase A	**Up-regulated under carbon limitation**	**Up-regulated in high glucose**
Glycolysis	Pgi1p, Phosphoglucose Isomerase	identified	**Up-regulated in high glucose**
Glycolysis	Pgm2p, Phosphoglucomutase	not identified	**Up-regulated in high glucose**
Gluconeogenesis	Fbp1p, Fructose-1,6-Bisphosphatase	not identified	not identified
Gluconeogenesis	Icl1p, Isocitrate Lyase	identified	not identified
Gluconeogenesis	Mls1p, Malate Synthase	**Up-regulated under carbon limitation**	not identified
Acetate Metabolism	Ach1p, Acetyl CoA Hydrolase	**Up-regulated under carbon limitation**	not identified
Mitochondrial ATPase	Atp2p, Mitochondrial ATP Synthase Subunit Beta	identified	not identified
Mitochondrial protein	Om45p, Mitochondrial Outer Membrane Protein	**Up-regulated under carbon limitation**	not identified
Heat Shock Protein	Hsp12p, Heat Shock Protein	not identified	**Down-regulated in high glucose**
Heat Shock Protein	Hsp26p, Heat Shock Protein	**Up-regulated under carbon limitation**	**Down-regulated in high glucose**
Heat Shock Protein	Hsp30p, Heat Shock Protein	not identified	not identified

Kolkman et al examined steady state expression of proteins in cells grown in two different conditions [9]. Carbon-limited medium contained 19 mM (NH_4_)_2_SO_4_ and 42 mM glucose. Nitrogen limited medium contained 7.5 mM (NH_4_)_2_SO_4_ and 330 mM glucose. Hxk1p, Ach1p, Mls1p, Om45p, and Hsp26p were up-regulated under carbon limitation, whereas Pma1p was down-regulated under carbon limitation. Pgi1p, Icl1p, and Atp2p were identified but showed no significant change in carbon limitation versus nitrogen limitation.

Pham et al examined steady state levels of proteins in cells grown in 120 g/L (normal) to 210 g/L and 300 g/L (high) concentrations of glucose for 68 hours [15]. Hxk1p, Pgi1p, and Pgm2p were up-regulated in high glucose, whereas Tef2p, Hsp12p, and Hsp26p were down-regulated by high glucose.

It has been reported that glucose induces mRNA levels 2–4 fold for more than 90% of the genes encoding ribosomal proteins [17]. When relative levels of ribosomal proteins were examined in our study, not all ribosomal subunits increase their abundance in response to glucose. For the key glycolytic enzyme phosphofructokinase, levels of Pfk1p and Pfk2p were similar whether cells were glucose starved or glucose replenished for 2 hours. We suggest that other mechanisms such as allosteric stimulation, protein modifications, or subunit assembly are likely to play more important roles in the regulation of the PFK activity.

One of the well-known effects of glucose regulation is the activation of the plasma membrane ATPase [4,56-58]. In this study, we have observed an increase in relative levels of Pma1p following the addition of glucose for 2 hours. For the same period of time, we did not find significant changes in the abundance of 6 out of the 15 subunits of the vacuole ATPase. The mitochondrial ATP synthase consists of 17 subunits. Of the 11 subunits that we have identified, 3 subunits reduced their abundance, while 8 other subunits did not change their levels. Atp1p and Atp2p are components of the catalytic F1 subunit which is known to be repressed by glucose [82]. Down-regulation of these subunits by glucose may be sufficient to cause a reduction in the ATPase activity. This is consistent with the finding that glucose-repressed cells contain fewer F1 particles in mitochondria as observed by electron microscopy of negatively stained mitochondria membranes [82]. It has been reported that many of the mitochondrial proteins such as Atp1p, Atp2p, Atp4p, Atp5p, Atp15p, Atp16p, and Atp20p are phosphorylated [99]. We suggest that protein degradation, protein modifications, allosteric inhibition, and subunit assembly may all contribute to the known decreased activities of mitochondrial enzymes that consist of multiple subunits.

Proteins that were observed to be down-regulated in the current experiments in response to glucose include several previously reported to be down-regulated such as Pgm2p, Fbp1p, Icl1p, Mls1p, Ach1p, Atp1p, Hsp12, Hsp26p, Hsp30p, and several proteins that were not previously reported to be down regulated, such as Hxk1p, Pgi1p, and Om45p. It is known that the transcription of ACH1, HXK1, HSP12, and HSP26 genes are repressed by glucose [24,28]. The levels of the corresponding proteins were also observed in the current work to be reduced in response to glucose addition.

A dramatic decline in protein levels in response to glucose was observed for the gluconeogenic enzymes Fbp1p and Icl1p. Malate synthase is involved in the gluconeogenesis/glyoxylate pathway. Transcription of MLS1 is repressed by glucose [16]. Furthermore, Mls1p activity is reduced following the addition of glucose [43]. Down-regulation of Fbp1, Icl1p, Mls1p, and Hsp30p by glucose was confirmed by Western blotting using cells that expressed GFP tagged proteins (Figure 3).

**Figure 5:**
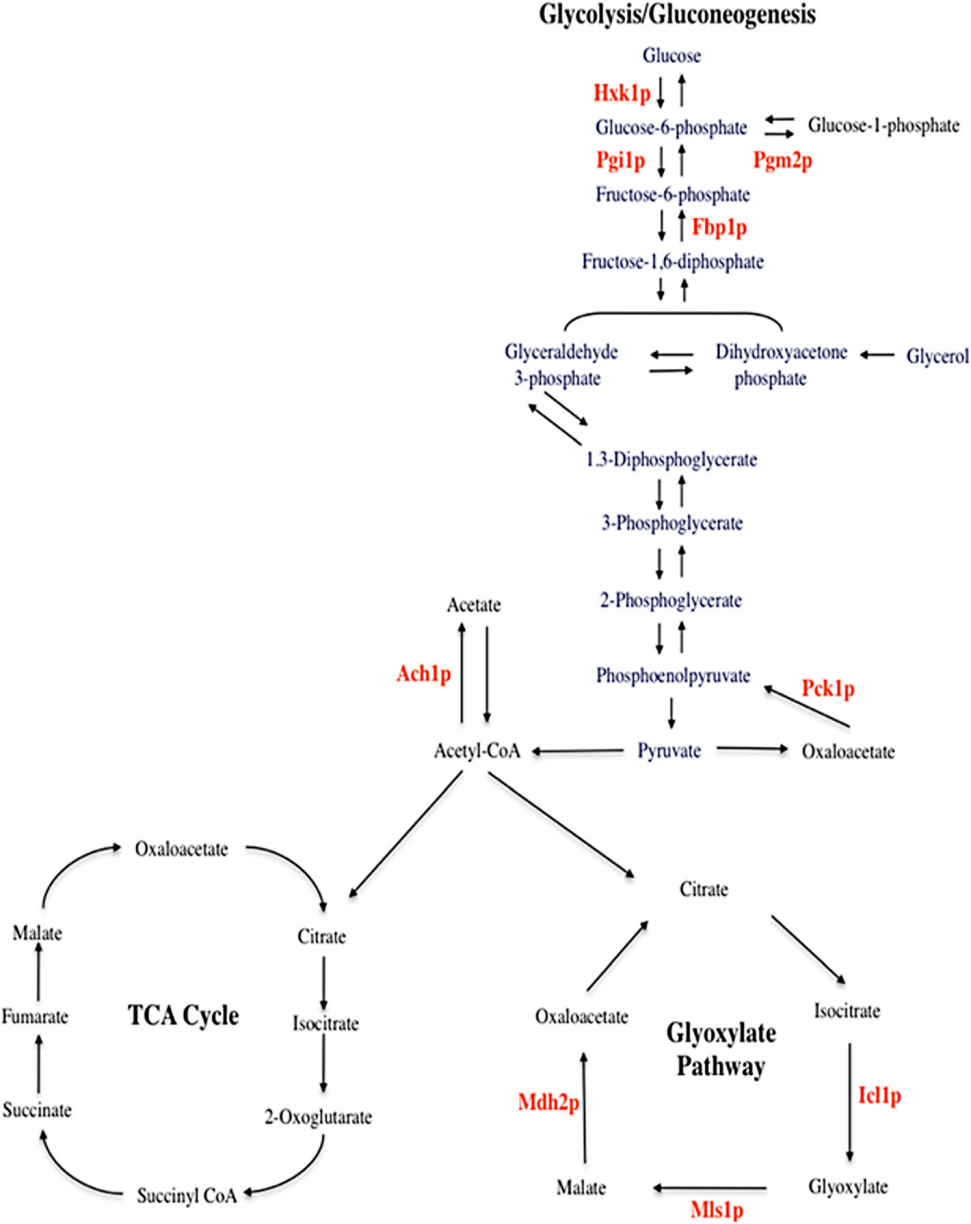
**Summary of glucose effects on proteins involved in glycolysis/gluconeogenesis and the TCA/glyoxylate cycle.** Glucose down-regulates Hxk1p, Pgi1p, and Pgm2p in the glycolytic pathway. Glucose also down-regulates Fbp1p in the gluconeogenic pathway, Icl1p and Mls1p in the TCA/glyoxylate cycle, and Ach1p in acetate metabolism. Mdh2p and Pck1p are known to be down-regulated by glucose and are included in this figure.

In summary, glucose up-regulates proteins involved in protein synthesis and nutrient uptake (Table 7). It also down-regulates small heat shock proteins, mitochondrial proteins, and proteins involved in gluconeogenesis (Table 7). For up-regulated proteins, glucose increases the abundance of several of the 40S and 60S ribosomal subunits, Tef2p, Tef4p, and Lia1p. Increased expression of these proteins may lead to an increase in protein synthesis. Glucose also up-regulates the plasma membrane ATPase, which is needed for the uptake of nutrients. In addition, glucose causes down-regulation of a number of proteins involved in glycolysis/gluconeogenesis, the TCA cycle, and the glyoxylate cycle (Figure 5). Although the significance of down-regulation of Hxk1p and Pgi1p is not clear at present, down-regulation of Pgm2p may lead to an increase in glycolysis. Pgm2p is the major enzyme that catalyzes the interconversion of glucose-6-phosphate and glucose-1-phosphate. Down-regulation of Pgm2p may reduce levels of glucose-1-phosphate required for the pentose pathway and the synthesis of glycogen, galactose, and trehalose. Consequently, more glucose-6-phosphate is available for the glycolytic pathway. Hence, down-regulation of Pgm2p may result in an increase in glycolysis. Glucose also causes the down-regulation of gluconeogenic enzymes, which leads to a decrease in gluconeogenesis. Down-regulation of Ach1p may cause a decline in the utilization of acetate when glucose is present. As Atp1p and Atp2p are components of the F1 catalytic subunits of the mitochondrial ATPase, decreased expression of these proteins may be sufficient to reduce the activity of the ATPase. The significance of down-regulation of these heat shock proteins in response to glucose is not presently known. One possibility is that some of these proteins need to be removed in order for cells to adapt to the new environments. For instance, Pma1p is negatively regulated by Hsp30p. Down-regulation of Hsp30p may remove the inhibitory effects of Hsp30p leading to the activation of Pma1p. Activated Pma1p may then stimulate the uptake of nutrients into the cells.

## Conclusions

Using the iTRAQ technique, several of the enzymes that are known to be regulated by glucose were identified in our study. Furthermore, we have also identified new glucose-regulated proteins that have not been previously reported to be regulated by glucose. Up-regulation of ribosomal proteins and proteins involved in protein translation may increase protein synthesis. Up-regulation of the plasma membrane ATPase may result in enhanced nutrient uptake. Down-regulation of glycolytic enzymes, gluconeogenic enzymes, and mitochondrial proteins may lead to changes in glycolysis, gluconeogenesis, and mitochondrial functions. These changes may be beneficial for cells to adapt to the new environments.

## Methods

### Cell Culture and Media

Yeast cells (BY4742*, MATα his3Δ1 leu2Δ0 lys2Δ0 ura3Δ0*) were grown in glucose-starved conditions in YPKG containing 1% yeast extracts, 2% peptone, 1% potassium acetate, and 0.5% glucose for 3 days and then shifted to YPD media containing 1% yeast extracts, 2% peptone, and 2% glucose for 2 hrs.

### iTRAQ Sample Preparation

Cells were lysed in 500 mM Hepes (pH 7.2), 50 mM MgSO4, 1 mM EDTA, and 1% SDS by vortexing with glass beads. A Bio Rad Protein Assay was used to measure the protein concentration in each sample. Protein from each sample (140 μg) was processed according to the Applied Biosystems iTRAQ kit, with the exceptions that iodoacetamide was used as the alkylating agent rather than MMTS and trypsin digestion was performed with Promega Sequencing Grade Trypsin (#: V511) overnight at 48°C. Protein from each sample (7 μg) was removed before and after addition of trypsin and digestion was confirmed by electrophoresis and Coomassie Blue staining. iTRAQ labeling was performed as follows: wild type *S. cerevisiae* cultures were starved of glucose for three days in YPKG medium and extracted peptides from duplicated samples were tagged with reagents 113 and 114. Similarly, duplicated samples from 3 day starved wild type *S. cerevisiae* cultures, which had been re-fed with glucose for 2 hours, were trypsin digested and tagged with reagents 115 and 116. These peptides were combined and subjected to 2D-LC separation and one MS/MS run.

### 2D-LC Separation and Mass Spectrometry

After iTRAQ labeling, the peptides from all separate labeling reactions were mixed together, dried down, and re-suspended in 10 ml of 10 mM ammonium formate, pH 3.6, in 20% acetonitrile/80% water. The combined peptides were separated by strong cation exchange separation (SCX) followed by reverse phase C18 nanoflow-LC separation, and spotted onto a stainless steel MALDI target plate, for a total of 370 spots per original SCX fraction.

SCX Separations were performed on a passivated Waters 600E HPLC system, using a 4.6 × 250 mm PolySULFOETHYL Aspartamide column (PolyLC, Columbia, MD) at a flow rate of 1 ml/min. Buffer A contained 10 mM ammonium formate, pH 3.6, in 20% acetonitrile/80% water. Buffer B contained 666 mM ammonium formate, pH 3.6, in 20% acetonitrile/80% water.

The gradient was Buffer A at 100% (0–22 minutes following sample injection), 0% → 40% Buffer B (22–48 min), 40% → 100% Buffer B (48–49 min), 100% Buffer B isocratic (49–56 min), then at 56 min switched back to 100% Buffer A to re-equilibrate for the next injection. The first 28 ml of eluant (containing all flow-through fractions) were combined into one fraction, then 14 additional 2-ml fractions were collected. All 15 of these SCX fractions were dried down completely to reduce volume and to remove the volatile ammonium formate salts, then resuspended in 9 μl of 2% (v/v) acetonitrile, 0.1% (v/v) trifluoroacetic acid and filtered prior to reverse phase C18 nanoflow-LC separation.

For the 2nd dimension separation by C18 reverse phase nanoflow LC, each SCX fraction was auto injected onto a Chromolith CapRod column (150 × 0.1 mm, Merck) using a 5 μl injector loop on a Tempo LC MALDI Spotting system (ABI-MDS/Sciex). Buffer C was 2% acetonitrile, 0.1% trifluoroacetic acid, and Buffer D was 98% acetonitrile, 0.1% trifluoroacetic acid.

The C18 elution gradient was 95% Buffer C/5% Buffer D (2 μl per minute flow rate from 0–3 min, then 2.5 μl per minute from 3–8.1 min), 5% Buffer D → 38% Buffer D (8.1-40 min), 38% Buffer D → 80% Buffer D (41–44 min), 80% Buffer D → 5% Buffer D (44–49 min) (initial conditions). Flow rate was 2.5 μl /min during the gradient, and an equal flow of MALDI matrix solution was added post-column (7 mg/ml recrystallized CHCA (a-cyano-hydroxycinnamic acid), 2 mg/ml ammonium phosphate, 0.1% trifluoroacetic acid, 80% acetonitrile).

The combined eluant was automatically spotted onto a stainless steel MALDI target plate every 6 seconds (0.6 μl per spot), for a total of 370 spots per original SCX fraction. Each MALDI target plate was analyzed in a data-dependent manner on either an ABI 5800 MALDI TOF-TOF or an ABI 4800 MALDI TOF-TOF. The MS spectra were taken from 5500 total MALDI Spots, averaging 500 laser shots per spot at laser power 2800. In a data-dependent manner, 12767 MS/MS spectra were taken from those same MALDI Spots, using up to 2600 laser shots per spectrum at laser power 3200, with CID gas at 1.2 to 1.3 × 10–6 Torr.

### Data analysis

All the data sets from the different plates were analyzed simultaneously; with protein identification and quantitation performed using the Paragon Algorithm [100] as implemented in ProteinPilot^TM^ 4.0 software (ABSciex). ProteinPilot^TM^ search parameters were set as follows: cysteine alkylation: iodoacetamide, ID focus: biological modifications, and search effort: thorough. We searched the combined spectra against the species-specific (*S. cerevisiae*) NCBI-nr database concatenated with a reversed “decoy” version of the same database plus 156 common lab contaminants identified by Keller et al [101]. The species-specific (*S. cerevisiae*) NCBI-nr database used was from Jan. 4, 2010 and contained 36621 protein sequences. The identification results from the Paragon Algorithm search were further filtered through the use of a very stringent Local False Discovery Rate (FDR) estimation calculated from the Proteomics System Performance Evaluation Pipeline (PSPEP) program [102-104]. This was used to reduce the potential number of false positive protein identifications. The FDR estimation is based on the number of hits obtained while simultaneously searching the “decoy” database, which is the exact reverse of each protein sequence [103,104]. For our list of identified proteins, we required protein IDs with a local FDR estimate of < 5%. By these stringent criteria, 591 proteins were identified. Detailed protein and peptide information and a full list of protein IDs are available as supplemental data, see Additional file 1: Table S1 and Additional file 2: Table S2, and from Proteomecommons.org Tranche using the following hash:nvaeDdOvABSNxenTjTpWroOkTW7hdoEZ9aejFVfpfKx0Vy + mqhf7YNweJsdZv3tezLkyobyFyXZ64wBCmMUIJK6MjwwAAAAAAAABxQ==. For the quantitative analyses, it is assumed that most proteins will not differ between two experimental conditions, and therefore the distribution of observed ratios should center near a median of 1.0 (a ratio of 1.0 would represent no relative change in that protein’s level between the two conditions being compared). With this assumption, a small data-dependent auto-bias correction was applied to each set of iTRAQ ratios such that the adjusted distribution of iTRAQ ratios observed had a median value of 1.0 (or 0 in log space), thus normalizing against any small discrepancies in total protein amount labeled or efficiency of individual iTRAQ labeling in the different samples. The relative change in protein ratios between three day low glucose conditions and glucose re-feeding for 2 hrs was accepted as significant for proteins with a p-value less than 0.05 as calculated by ProteinPilot^TM^ 4.0 based on the ratios of each identified peptide in at least three out of four comparisons and proteins with more than 1 peptide identified with at least 95% of confidence. A one-sample Student's *t*-test was used to confirm that each protein's set of ratios for all four comparisons was significantly different than 1. From the identifiers (gi numbers) obtained from searching against the NCBI-nr database, we used the Gene Ontology Slim Mapper program available in the *Saccharomyces* Genome Database (Stanford University) to search for the function and subcellular location for each protein. The overlap in identified proteins between other MS-based proteomics studies was compared using the online tool BioVenn [105]. The alignment of primary amino acid sequences of 12 proteins that are down-regulated by glucose is also available, see Additional file 3.

### Western blotting

Cells that expressed Lia1p-GFP, Fbp1p-GFP, Icl1p-GFP, Mls1p-GFP, and Hsp30p-GFP were purchased from Invitrogen. These cells were starved of glucose for three days and re-fed with glucose for 0, 2, and 3 hours. Total lysates were prepared and proteins were separated by SDS-PAGE. Proteins were transferred to nitrocellulose membrane and blotted with anti-GFP antibodies (Abcam) followed by HRP conjugated goat anti-rabbit antibodies (GE Healthcare). Proteins were detected with the ECL kit from PerkinElmer, Inc.

## Abbreviations

iTRAQ: Isobaric Tags for Relative and Absolute Quantification; MALDI: Matrix-assisted laser desorption/ionization; FDR: False Discovery Rate; MS: Mass Spectroscopy; SCX: Strong Cation Exchange.

## Competing interests

The authors declare that they have no competing interests.

## Authors’ contributions

BJG participated in sample preparations, data collection, data analysis, and drafting of the manuscript. BAS was involved in data collection, data interpretation, and the drafting of the manuscript. HLC participated in the design of the experiments and the drafting of the manuscript. All three authors approved the order of the authorship. All authors read and approved the final manuscript.

## Supplementary Material

Additional file 1: Table S1 Proteins identified with a local FDR < 5%. Click here for file

Additional file 2: Table S2 Peptide summary information for iTRAQ experiment. Click here for file

Additional file 3 Alignment of primary amino acid sequences of 12 proteins that are down-regulated by glucose. Click here for file
